# Study on obtaining bacterial cellulose by *Komagataeibacter xylinus* in co-culture with lactic acid bacteria in whey

**DOI:** 10.1007/s00253-025-13582-3

**Published:** 2025-08-21

**Authors:** Justyna Płoska, Monika Garbowska, Iwona Ścibisz, Lidia Stasiak-Różańska

**Affiliations:** Department of Food Technology and Assessment, Institute of Food Sciences, Warsaw University of Life Sciences - SGGW, Nowoursynowska Street 166, 02-787 Warsaw, Poland

**Keywords:** Bacterial cellulose, *Komagataeibacter xylinus*, Lactic acid bacteria, *Lactobacillus*, Lactic acid, Acid whey, Co-culture

## Abstract

**Abstract:**

The use of acid whey as a medium is an innovative approach to bacterial cellulose (BC) biosynthesis in co-cultures of acetic acid bacteria with lactic acid bacteria. The aim of this study was to evaluate the possibility of obtaining BC in acid whey by co-culturing *K. xylinus* with selected strains of lactic acid bacteria and comparing the properties of this biopolymer with BC obtained in *K. xylinus* monoculture. The *K. xylinus* + *Lb. acidophilus* co-culture yielded 2.19 g·L^−1^ of BC, which was 125% more than the *K. xylinus* monoculture. Additionally, *K. xylinus* in co-culture with *Lb. acidophilus* increased the degradation temperature of BC to 361 °C compared to 303 °C for BC obtained in monoculture. The BC obtained in the co-cultures showed better mechanical properties. BC obtained in co-culture with *Lb. delbrueckii* showed more than twice the Young’s modulus than BC from monoculture. Moreover, strain at break BC from co-culture with *Lb. acidophilus* and stress at break BC from co-culture with *Lb. helveticus* were 72% and 54% higher, respectively, than BC obtained from monoculture *K. xylinus.* In this study, it was shown that conducting acetic-lactic co-cultures increased the efficiency of BC biosynthesis and improved its properties. Moreover, this study has shown that acid whey is a sufficient and complete substrate for obtaining BC. Results presented in this paper indicate new possibilities for the management of this side product.

**Key points:**

• *The K. xylinus + Lb. acidophilus co-culture produced 125% more cellulose than the monoculture.*

• *High lactic acid content and low pH of acid whey enhance cellulose biosynthesis.*

• *Acetic acid-lactic acid co-cultures improved the mechanical properties of cellulose.*

**Supplementary Information:**

The online version contains supplementary material available at 10.1007/s00253-025-13582-3.

## Introduction

Acid whey (AW) is a by-product of the dairy industry, obtained during acidic cheese production. It contains nutrients such as lactose, whey proteins, organic acids (lactic acid, citric acid), macro- and micronutrients, and B vitamins (B_1_, B_2_, B_5_) (Panesar et al. 2007). Non-protein nitrogen compounds (urea, uric acid) and phosphorus compounds are also present in AW, making a risk of eutrophication if AW enters the environment. The presence of organic compounds makes AW a by-product difficult to manage and harmful for the natural environment (Kazimierowicz et al. 2022). Therefore, it is important to find a solution to manage AW and reduce its adverse impact on the surrounding environment. In turn, the diversity of nutrients in AW means that this by-product might be a medium for the cultivation of microorganisms. The use of AW for the biosynthesis of new compounds fits into the framework of a circular economy (Lappa et al. 2021). In addition, some microbial metabolites can add value and enrich food products, thanks to which they can be considered an element of fulfillment of Sustainable Development Goal 12 (Goal 12: Ensure sustainable consumption and production patterns). So far, AW has been used for the microbial biosynthesis of lactic acid (LA), bioethanol, fatty acids, and polyhydroxyalkanoates, among others (Murari et al. 2019; Sampaio et al. 2020; Lappa et al. 2021; Bosco et al. 2021; Uysal and Hamamcı 2021).

An interesting biopolymer of microbial origin that can be made from AW is bacterial cellulose (BC). BC is a natural exopolysaccharide made of linear β−1,4-glucan chains linked by hydrogen bonds, van der Waals forces, and hydrophobic interactions (Płoska et al. 2023b). Selected species of acetic acid bacteria (AAB), in particular *Komagataeibacter xylinus*, are considered to be the main producers of BC (Blanco Parte et al. 2020). BC is characterized by biodegradability, high purity, high mechanical strength, liquid and gas permeability, ability to adsorb various compounds, and biocompatibility with living tissues (Faria et al. 2022; Kumaravel et al. 2025). In the food industry, BC can be used as a stabilizer (e.g., Pickering emulsion gels as fat replacers in ice cream), emulsifier (nanoemulsion of cinnamon oil), fat substitute (e.g., in ice cream), and in food packaging (e.g., bacterial cellulose/tomato puree edible films in multicomponent foods, BC-based material for cheese packaging) (Guo et al. 2018; Razavi et al. 2020; Freitas et al. 2022; Gao et al. 2023; Płoska et al. 2024). Despite the unique properties of BC and a number of potential applications, this polymer is still not produced on a large scale. Obtaining BC on an industrial scale is expensive, mainly due to the high prices of media required for AAB growth. Therefore, it is reasonable to look for cheaper alternatives, e.g., replacing typical culture media with inexpensive industrial waste and by-products.

In our previous work, we obtained in AW approx. 3 g·L^−1^ BC, which was approx. 2 g·L^−1^ less compared to the classic medium (Płoska et al. 2023a). The research was continued because an interesting dependency was observed. The highest BC mass was obtained in AW, in which the content of organic acids (expressed as LA) was the lowest after 14 days of culture. AW contains mainly LA and citric acid (CA) (Menchik et al. 2019). The positive effect of LA on AAB growth has been documented several times (Matsuoka et al. 1996; Cielecka et al. 2021a; Płoska et al. 2023a). According to these reports, LA acts as an accelerator of tricarboxylic acid (TCA) cycle, stimulating the growth of AAB cells, which in turn translates into increased efficiency of BC production. Moreover, it was found that LA, when converted into pyruvate, generates additional energy, which positively affects the BC biosynthesis (Cielecka et al. 2021a). Lactic acid bacteria (LAB) are usually responsible for converting lactose to LA (Ayivi et al. 2020). Co-cultivation of *K. xylinus* with LAB for BC biosynthesis may provide many benefits; for example, due to the diverse metabolic pathways of microorganisms, the product of the metabolism of one strain can also be a substrate in the metabolism of another (Antolak et al. 2021). LAB assimilate lactose present in AW, which is difficult to absorb by most AAB, and convert it into LA. Another potential benefit of co-cultures may be the fact that some LAB produce exopolysaccharides (EPS) that can positively affect the chemical-physical and mechanical properties of BC (Liu and Catchmark 2019). Recently, scientific reports have begun to appear raising the possibility of using co-cultures as a way to increase the efficiency of obtaining BC and/or improving its properties. For example, Brugnoli et al. (2023a, b) carried out a study where a co-culture system of two AAB strains and two LAB strains was developed to produce BC and hyaluronic acid composites. As a result, the co-cultures yielded more BC than the AAB monocultures, and the obtained composites had different properties with application potential in cosmetics and pharmaceuticals. However, there are still many unknowns in this field, and there is much to be explored. Thus, there is a need to fill the gap concerning, among other things, the selection of strains and composition of the medium (Huang et al. 2024; Qiao et al. 2024; Mollica et al. 2024).

The novel approach of this work is that instead of typical microbiological media (used in most of the works in this field, e.g., Hestrin-Schramm), we used only AW—a side product from the dairy industry as a medium for co-cultures and BC synthesis. Nowadays, AW is a particularly concerning environmental problem. The management of this side product is also the subject of many research. There is a lack of information in the scientific literature on the use of AW as a medium for BC production in acetic-lactic co-culture. Therefore, our research focuses on this aspect.

In this study, we assumed that LA produced by LAB promotes the metabolic pathway of BC biosynthesis in AAB. Moreover, we hypothesized that co-culture of *K. xylinus* with LAB will increase BC efficiency because LAB, by breaking down lactose, will increase the availability of easily digestible simple sugar (glucose), and *K. xylinus* will be able to synthesize BC more efficiently. The use of LAB species can help explore the different interactions occurring between bacteria in co-culture, as well as the potential mechanisms of BC biosynthesis intensification and how this affects its properties. The aim of the research was to test the possibility of increasing the efficiency of BC production in acetic-lactic co-culture in AW. In addition, the impact of the type of culture (monoculture *vs* co-culture) on the chemical, thermal, and mechanical properties of BC was examined.

## Materials and methods

### Microbial strains

In this study, one strain of AAB was used: *Komagataeibacter xylinus* UMCC 2756 (Unimore Microbial Culture Collection*,* University of Modena and Reggio Emilia, Italy) and three strains of LAB were used: *Lactobacillus acidophilus* ATCC 4356 (American Type Culture Collection, USA), *Lactobacillus helveticus* LhB01 (Chr. Hansen, Ltd., Czosnów, Poland), and *Lactobacillus delbrueckii* subsp. *lactis* ATCC 4797 (American Type Culture Collection, USA).

### Co-culture preparation

Preinoculum of *K. xylinus* was multiplied in YPM medium (composition: 5 g·L^−1^ yeast extract, 3 g·L^−1^ peptone, 25 g·L^−1^ mannitol, pH 5.75) for 96 h at 30 °C with shaking (120 rpm), then seeded on YPM-agar slants and cultured for 96 h at 30 °C. Preinoculum of *Lb. acidophilus*, *Lb*. *delbrueckii*, and *Lb. helveticus* was multiplied in De Man, Rogosa, and Sharpe liquid medium (MRS, Merck, Poland) for 96 h at 30 °C under stationary conditions, then seeded on MRS slants and cultured for 96 h at 30 °C.

*K. xylinus* inoculum was prepared by taking colonies from agar slants and suspending them in saline to obtain a turbidity comparable to McFarland standard turbidity of 2.0 (approximately 8.78 log CFU mL^−1^). LAB inoculum was prepared similarly. Acid whey (AW) (Laktopol Dairy Plant, Łosice, Poland), as a BC production medium, was reconstituted from powder: 70 g·L^−1^, pH 4.8, and then autoclaved at 118 °C for 15 min. Sterile AW was inoculated with 10% v/v inoculum (5% *K. xylinus* + 5% LAB strain) (Płoska et al. 2024). The ratio of *K. xylinus* to LAB bacteria in co-culture was 1:1 (Jiang et al. 2023).

Three variants of acetic-lactic co-cultures were made:

1. *K. xylinus* + *Lb. acidophilus* ATCC 4356
2. *K. xylinus* + *Lb. delbrueckii* subsp. *lactis* ATCC 4797
3. *K. xylinus* + *Lb. helveticus* LhB01

In addition, single cultures (monocultures) of all LAB and *K. xylinus* strains were conducted in AW for 14 days at 30 °C.

### Obtaining BC in mono- and co-cultures and preparing it for further analyses

During culture, a network of polymer fibers was formed on the surface of the AW observed as a shiny, flexible membrane. The amount of BC produced in *K. xylinus* monoculture and co-cultures was evaluated at the beginning of culture (T0) and after 3, 6, 9, 12, and 14 days. Whey pH was measured at the same time points. BC obtained in *K. xylinus* monoculture and all co-culture variants was purified from bacterial cells and residual medium by incubation in 0.1 M NaOH at 95 °C for 5 h. BC was neutralized with 6% acetic acid, rinsed with distilled water, and sterilized at 121 °C for 20 min. Next, all BC sheets were placed on the sterile paper and dried at 45 °C for around 5 h ± 30 min until to establish a constant mass (Płoska et al. 2023a).

### Determination of sugars and organic acids

Lactose, galactose, and glucose, as well as acetic acid (AA), CA, gluconic acid (GA), and LA, were determined in the AW by high-performance liquid chromatography (HPLC). Samples were centrifuged at 10,000 rpm for 10 min (MPW-350R; MPW Med. Instruments, Warsaw, Poland) and the supernatant was collected. The appropriately diluted supernatant was filtered through a 0.45-μm nylon syringe filter. The analysis of sugars in media followed a modified procedure based on the method described by Usenik et al. (2008) and Elghali et al. (2005). Chromatographic analyses were carried out using a Shimadzu Prominence HPLC system (Kyoto, Japan), comprising an LC-20AD pump, autosampler (SIL-20A HT), refractive index detector (RID-10A), column oven (CTO-10ASVP), degasser (DGU-20A5R), and LABSolution data collection software. Separation was executed on a RezexTM RCM-Monosaccharide Ca^+^ column (300 × 7.8 mm) (Phenomenex, USA) at 65 °C. The mobile phase used was Milli-Q water eluted with a flow rate of 0.7 mL·min^−1^.

The analysis of organic acid in media was performed according to the procedure described by Fernandez-Garcia and McGregor (1994) with minor modifications. Organic acid analysis was performed using the aforementioned Shimadzu HPLC system, equipped with a diode array detector (SPD M20A). Cosmosil 5C18-PAQ (4.6 mm × 150 mm) column (Waters, Etten-Leur, The Netherlands) was used for the separation of organic acid. The column temperature was maintained at 20 °C, and the mobile phase consisted of 20 mmol phosphoric acid with a flow rate of 1.0 mL·min^−1^. A 20 µL injection volume was used, and organic acid peaks were detected at 210 nm. The organic acids were identified by comparing their retention times and UV–Vis spectral characteristics to those of commercial standards. Lactose, glucose, galactose, and GA were obtained from Sigma-Aldrich with purities of 97–99%. CA, AA, and L(+)-LA were purchased from Merck KGAA (Darmstadt, Germany).

### Assessment of exopolysaccharides production capacity

The ability of LAB to produce EPS was evaluated in MRS medium and in AW. Inoculum was prepared by suspending bacterial colonies in saline to obtain a turbidity comparable to McFarland standard turbidity of 2.0 (approximately 8.78 log CFU mL^−1^). In co-cultures, the ratio of AAB to LAB cells was 1:1. The analysis of EPS production was performed according to the procedure described by Liu and Catchmark (2019) and Ma’unatin et al. (2020) with minor modification. After finishing the cultivation, the medium was heated to 100 °C for 15 min to denature EPS-degrading enzymes. After cooling, the samples were centrifuged (10,000 rpm, 4 °C, 15 min). Then, EPS present in the supernatants was precipitated by adding 3 volumes of cold ethanol (96%) and left overnight at 4 °C, then centrifuged again. The resulting EPS precipitate was freeze-dried and weighed on an analytical balance (Liu and Catchmark 2019; Ma’unatin et al. 2020).

### Characterization of bacterial cellulose

#### Scanning electron microscope analysis

BC surface morphology and cross section were analyzed using the Jeol ISM-IT500HR INTOUCHSCOPE (JEOL Europe SAS) scanning electron microscope. BC films were attached to carbon tape and coated with gold. The surface structure was analyzed at a magnification of × 15,000 and the cross section was analyzed at a magnification of × 3000– × 3500 at an accelerating voltage of 15 kV and 5 kV for the surface and cross section, respectively. The average diameter of BC fibers was measured using ImageJ software (National Institute of Health, Bethesda, MD, USA) on 40 randomly selected fibers on SEM images.

### Attenuated total reflectance-Fourier transform infrared characterization

ATR-FTIR spectra were acquired by means of a Nicolet iS20 spectrophotometer (Thermo Scientific, Waltham, MA, USA). The spectra were collected in the range of 600–4000 cm^−1^, at a resolution of 4 cm^−1^, 32 scans. The ATR-FTIR air background spectrum was collected every 30 min. Spectra were normalized to 1.0 at 1030 cm^−1^ (C-O–H stretching vibration). Baseline corrections were obtained on Omnic Software (Thermo Scientific).

### Thermogravimetric analysis

To study the thermal properties of BC, thermogravimetric analysis (TGA) and derivative thermogravimetric analysis (DTG) were performed on a Mettler Toledo™ TGA/DSC1 apparatus. The analysis was carried out from room temperature (25 °C) to 600 °C, with a heating rate of 10 °C·min^−1^ and in a nitrogen atmosphere with a flow rate of 50 ml·min^−1^.

### Mechanical properties

The tensile properties of BC were performed using Instron 3365 (Instron, USA) machine at room temperature (25 °C). Specimens of 30 mm length and 5 mm width were stretched at a rate of 1.5 mm·min^−1^ until fracture. The thickness of the nanocomposites was measured using an electronic MI21 micrometer (MTS, Slovakia). Strain at break (ε) was calculated as ΔL/L_0_ where L_0_ is the initial length of a sample between clamps and ΔL is the increased length from L0. Stress at break (δ) was calculated by F/A, where F is the force applied in Newtons and A is the area of the cross-section of the sample. The apparent Young’s modulus (E) was defined by the slope of the linear region of the strain–stress curve during the stretching stage. All measurements were performed in ten repeats, and the average value was recorded.

### Statistical analyses

All experiments were conducted in triplicate (unless otherwise noted) and results are presented as mean ± standard deviation (SD). One-way analysis of variance (ANOVA) was performed using Statistica 13.1 software (Dell Sp. z o.o., Warsaw, Poland). The Tukey test was applied to compare the significance of differences between mean values at a significance level of *α* = 0.05.

## Results

### The efficiency of BC biosynthesis in mono- and co-cultures

**Fig. 1:**
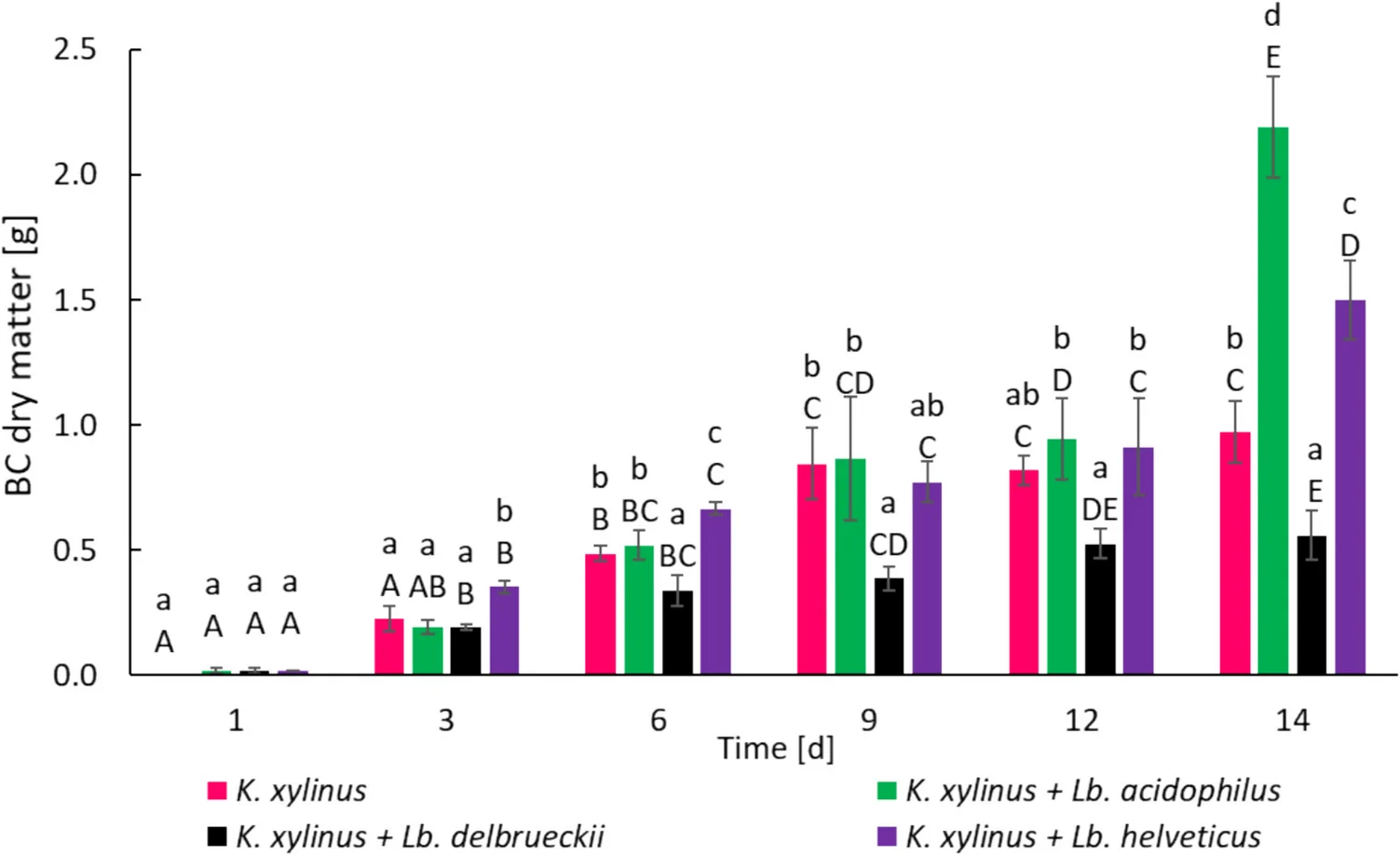
BC dry matter obtained during monoculture of *K. xylinus* and acetic-lactic co-cultures; ^a–d^Means marked with the same lowercase letters are not statistically significantly different at α = 0.05 within time. ^a–e^Means marked with the same uppercase letters are not statistically significantly different at α = 0.05 within one culturing variant conducted during 14 days

The dry weight of BC obtained in the *K. xylinus* monoculture and co-cultures is shown in Fig. 1.

BC was observed after just 1 day of culture in the co-cultures, whereas in the monoculture *K. xylinus*, BC appeared only after 3 days. Over time, the BC mass increased in all cultures, except for the *K. xylinus* + *Lb. delbrueckii* co-culture. Statistically significant differences (*p* < 0.05) in BC dry matter (dm) were observed even after 3 and 6 days, with the highest BC production occurring in the *K. xylinus* + *Lb. helveticus* co-culture (0.35 g·L^−1^ and 0.67 g·L^−1^, respectively). After 12 days of cultivation, the dm of BC in the *K. xylinus* + *Lb. acidophilus* and *K. xylinus* + *Lb. helveticus* co-cultures was comparable to the mass of BC from the *K. xylinus* monoculture and amounted to 0.95 g·L^−1^, 0.91 g·L^−1^, and 0.82 g·L^−1^, respectively. However, after 14 days, the highest BC yield was obtained in the *K. xylinus* + *Lb. acidophilus* and *K. xylinus* + *Lb. helveticus* co-cultures (2.19 g·L^−1^ and 1.5 g·L^−1^, respectively) compared to the monoculture, where approximately 1 g·L^−1^ was produced. This constituted 125% and 54% more BC, respectively, than in the *K. xylinus* monoculture. The increase in the BC mass in two of the three studied acetic-lactic co-cultures confirms the conclusions of other researchers that different strain combinations in co-cultures have varying effects on the BC biosynthesis (Jiang et al. 2023).

### Changes in sugar content in AW in mono- and co-cultures

**Fig. 2:**
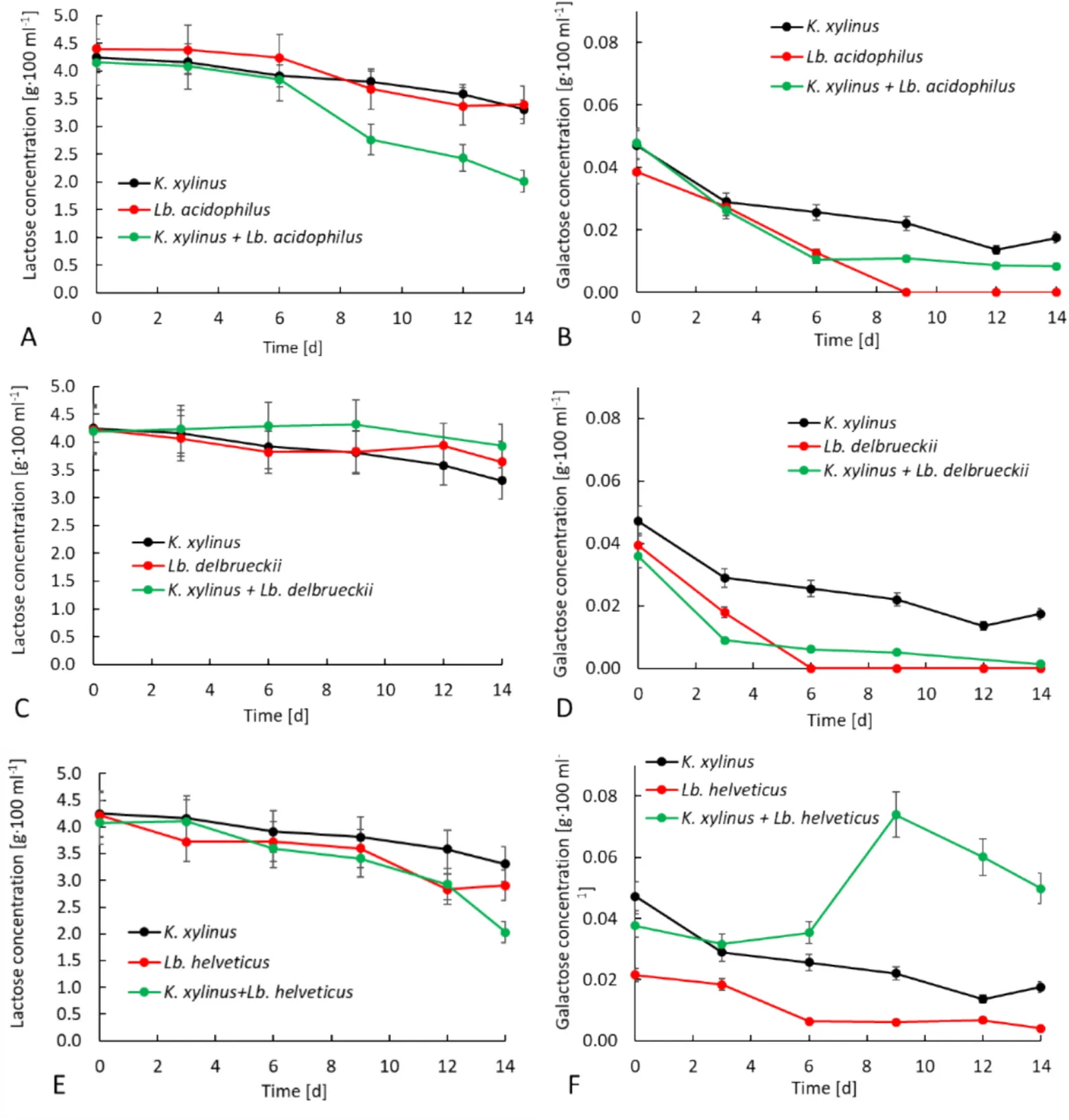
Lactose (**A**, **C**, **E**) and galactose (**B**, **D**, **F**) content in *K. xylinus* monoculture and co-cultures

Chromatographic analysis showed no glucose in the whey during the 14 days of culture. The content of lactose and galactose in AW with the different bacterial variants is shown in Fig. 2.

The lactose content in AW before the start of the cultivation (T_0_) was similar across most variants amounting to 4.2 g·100 mL^−1^. A similar lactose content in the whey medium was noted in previous studies by Revin et al. (2018). The lactose content in the medium decreased over time. The largest utilization of lactose after 14 days of cultivation occurred in the *K. xylinus* + *Lb. acidophilus* and *K. xylinus* + *Lb. helveticus* co-cultures (2.2 g·100 mL^−1^ and 2.1 g·100 mL^−1^, respectively) (Fig. 2A and E). This was most likely due to the highest BC biosynthesis in these co-cultures (Fig. 1), high LA production (Table 2), and the consumption of a carbon source to ensure the basic metabolic functions of the cells. In the *K. xylinus* monoculture, lactose consumption was 0.9 g·100 mL^−1^, which was reflected in the dm of BC, which after 14 days of cultivation was significantly (*p* < 0.05) lower than in the *K. xylinus* + *Lb. acidophilus* and *K. xylinus* + *Lb. helveticus* co-cultures (Fig. 1)*.*

#### Changes in organic acid content in AW in mono- and co-cultures

Table 1 shows the pH of whey during monoculture and co-culture.

**Table 1:** Changes of pH of media during BC biosynthesis in monocultures and co-cultures

Strains	Time (d)					
	0	3	6	9	12	14
*K. xylinus*	4.80 ± 0.02^c^	5.28 ± 0.06^d^	4.26 ± 0.04^b^	4.06 ± 0.14^ab^	3.90 ± 0.16^a^	3.84 ± 0.19^a^
*Lb. acidophilus*	4.82 ± 0.01^e^	4.51 ± 0.06^d^	4.09 ± 0.02^c^	3.90 ± 0.01^b^	3.75 ± 0.02^a^	3.73 ± 0.01^a^
*Lb. delbrueckii*	4.82 ± 0.01^c^	4.65 ± 0.03^b^	4.53 ± 0.03^a^	4.54 ± 0.01^a^	4.57 ± 0.02^a^	4.58 ± 0.03^a^
*Lb. helveticus*	4.82 ± 0.01^f^	4.43 ± 0.01^e^	4.11 ± 0.01^d^	4.04 ± 0.02^c^	3.91 ± 0.03^b^	3.85 ± 0.02^a^
*K. xylinus* + *Lb. acidophilus*	4.80 ± 0.01^c^	4.21 ± 0.03^b^	3.95 ± 0.10^a^	3.81 ± 0.12^a^	3.81 ± 0.08^a^	3.83 ± 0.02^a^
*K. xylinus* + *Lb. delbrueckii*	4.79 ± 0.02^a^	4.96 ± 0.01^b^	5.85 ± 0.08^c^	5.92 ± 0.03^c^	5.95 ± 0.01^c^	5.96 ± 0.02^c^
*K. xylinus* + *Lb. helveticus*	4.82 ± 0.06^b^	4.50 ± 0.10^b^	3.96 ± 0.11^a^	3.77 ± 0.22^a^	3.71 ± 0.11^a^	3.59 ± 0.16^a^

^a–f^Means marked with the same superscript letters in the same row do not differ statistically significantly at *α* = 0.05

At the beginning of bacterial growth (T_0_), the pH of the monocultures and acetic-lactic co-cultures media were comparable, averaging 4.82. During the culture incubation, the progressive fermentation and BC biosynthesis led to changes in the active acidity of the media. In the *K. xylinus* monoculture, the pH increased after 3 days of cultivation to 5.28, and then systematically decreased to 3.84 after 14 days of cultivation. Such pH changes could be due to the assimilation of LA contained in AW at the start of culture (Table 2, LA concentration at T0 = 0.53 g·100 mL^−1^, LA concentration at T3 = 0.44 g·100 mL^−1^), which resulted in an increase in pH (day 3) followed by the production of acidic, pH-lowering metabolites in the subsequent days of culture. In the case of the *K. xylinus* + *Lb. acidophilus* and *K. xylinus* + *Lb. helveticus* co-cultures, a systematic pH decrease was observed during the culture, from pH 4.80 and pH 4.82 to pH 3.83 and pH 3.59, respectively. This progressive acidification of the medium occurred over a 14-day cultivation period and was accompanied by the highest BC production observed in this study (Fig. 1). We assume that the ability of *Komagataeibacter* spp. and *Lactobacillus* spp. to tolerate low pH conditions enabled the cultures to proceed efficiently and produced BC even as the pH approached 3.6. To sum up, as the cultivation progressed, the acidity of the medium increased, while the yield of BC also increased. The increase in acidity in these co-cultures was likely caused by the increased production of LA by *Lb. acidophilus* and *Lb. helveticus* (Table 2). This is supported by the significant decrease in the pH of whey in the monocultures of these bacteria. It was also observed that in the co-cultures, the pH stabilized and did not change significantly after 6 days of cultivation. In the *K. xylinus* + *Lb. delbrueckii* co-culture, an increase in pH was observed during cultivation. After 3 days, the pH was higher than at the beginning of cultivation, and after 9 days, the pH reached 5.92 and remained at a similar level until the end of incubation. One possible explanation for this effect is the slight decrease in pH from 4.82 to 4.58 observed in the *Lb. delbrueckii* monoculture, suggesting a lower capacity for LA production by *Lb. delbrueckii* compared to other LAB (Table 2) (Giraffa et al. 2004). The content of individual organic acids in AW during monoculture and co-culture is shown in Table 2.

**Table 2:** Organic acid content during BC biosynthesis in monocultures and co-cultures (g·100 mL^−1^)

Strain	Organic acid	Time (d)					
		0	3	6	9	12	14
		(g·100 mL^−1^)					
*K. xylinus*	AA	0.00 ± 0.000	0.00 ± 0.000	0.00 ± 0.000	0.00 ± 0.000	0.00 ± 0.000	0.00 ± 0.000
	CA	0.07 ± 0.017	0.06 ± 0.007	0.04 ± 0.031	0.06 ± 0.009	0.05 ± 0.003	0.06 ± 0.010
	GA	0.08 ± 0.029	0.05 ± 0.009	0.03 ± 0.019	0.05 ± 0.026	0.03 ± 0.024	0.07 ± 0.039
	LA	0.53 ± 0.100	0.44 ± 0.051	0.64 ± 0.029	0.78 ± 0.083	0.89 ± 0.129	1.04 ± 0.039
*Lb. acidophilus*	AA	0.00 ± 0.000	0.00 ± 0.000	0.00 ± 0.000	0.00 ± 0.000	0.00 ± 0.000	0.00 ± 0.000
	CA	0.06 ± 0.009	0.06 ± 0.005	0.05 ± 0.011	0.04 ± 0.003	0.04 ± 0.014	0.05 ± 0.007
	GA	0.07 ± 0.009	0.03 ± 0.018	0.04 ± 0.020	0.03 ± 0.018	0.03 ± 0.017	0.04 ± 0.023
	LA	0.50 ± 0.057	0.61 ± 0.097	0.91 ± 0.146	0.88 ± 0.045	1.07 ± 0.218	1.25 ± 0.159
*Lb. delbrueckii*	AA	0.00 ± 0.000	0.00 ± 0.000	0.00 ± 0.000	0.00 ± 0.000	0.00 ± 0.000	0.00 ± 0.000
	CA	0.06 ± 0.008	0.06 ± 0.003	0.08 ± 0.006	0.06 ± 0.008	0.06 ± 0.005	0.06 ± 0.013
	GA	0.05 ± 0.025	0.03 ± 0.005	0.03 ± 0.000	0.03 ± 0.000	0.03 ± 0.001	0.03 ± 0.008
	LA	0.50 ± 0.039	0.55 ± 0.067	0.74 ± 0.051	0.54 ± 0.057	0.60 ± 0.036	0.52 ± 0.107
*Lb. helveticus*	AA	0.00 ± 0.000	0.00 ± 0.000	0.00 ± 0.000	0.00 ± 0.000	0.00 ± 0.000	0.00 ± 0.000
	CA	0.08 ± 0.018	0.06 ± 0.003	0.06 ± 0.003	0.06 ± 0.010	0.07 ± 0.017	0.06 ± 0.004
	GA	0.08 ± 0.011	0.06 ± 0.007	0.06 ± 0.014	0.05 ± 0.009	0.06 ± 0.013	0.06 ± 0.004
	LA	0.47 ± 0.072	0.55 ± 0.069	0.71 ± 0.030	0.84 ± 0.119	1.02 ± 0.134	0.94 ± 0.066
*K. xylinus* + *Lb. acidophilus*	AA	0.00 ± 0.000	0.00 ± 0.000	0.00 ± 0.000	0.00 ± 0.000	0.00 ± 0.000	0.00 ± 0.000
	CA	0.05 ± 0.000	0.05 ± 0.009	0.05 ± 0.014	0.04 ± 0.012	0.04 ± 0.009	0.04 ± 0.008
	GA	0.07 ± 0.012	0.03 ± 0.004	0.04 ± 0.008	0.04 ± 0.016	0.05 ± 0.015	0.06 ± 0.006
	LA	0.47 ± 0.017	0.69 ± 0.114	0.99 ± 0.191	0.96 ± 0.115	1.06 ± 0.188	0.85 ± 0.142
*K. xylinus* + *Lb. delbrueckii*	AA	0.00 ± 0.000	0.00 ± 0.000	0.06 ± 0.024	0.06 ± 0.006	0.06 ± 0.002	0.07 ± 0.001
	CA	0.06 ± 0.003	0.06 ± 0.009	0.00 ± 0.000	0.00 ± 0.000	0.00 ± 0.000	0.00 ± 0.000
	GA	0.08 ± 0.006	0.06 ± 0.044	0.04 ± 0.009	0.04 ± 0.003	0.04 ± 0.008	0.05 ± 0.016
	LA	0.49 ± 0.010	0.52 ± 0.071	0.41 ± 0.048	0.31 ± 0.049	0.30 ± 0.035	0.28 ± 0.038
*K. xylinus* + *Lb. helveticus*	AA	0.00 ± 0.000	0.00 ± 0.000	0.00 ± 0.000	0.00 ± 0.000	0.00 ± 0.000	0.02 ± 0.004
	CA	0.06 ± 0.005	0.06 ± 0.002	0.05 ± 0.037	0.06 ± 0.014	0.05 ± 0.013	0.03 ± 0.012
	GA	0.06 ± 0.007	0.09 ± 0.012	0.08 ± 0.025	0.12 ± 0.051	0.08 ± 0.021	0.08 ± 0.017
	LA	0.45 ± 0.046	0.60 ± 0.069	0.93 ± 0.140	1.21 ± 0.126	1.36 ± 0.139	1.47 ± 0.155

*AA* acetic acid, *CA* citric acid, *GA* gluconic acid, *LA* lactic acid, mean ± standard deviation.

AAB produce acetic acid through a two-step oxidation reaction of ethanol to acetaldehyde, which is then converted to acetic acid (He et al. 2022). Since there is no ethanol in AW, AA was not detected in *K. xylinus* monoculture or the *K. xylinus* + *Lb. acidophilus* co-culture as expected. However, in the *K. xylinus* + *Lb. delbrueckii* co-culture, AA was observed after 6 days of culture, and its concentration remained constant at 0.06–0.07 g·100 mL^−1^. In the *K. xylinus* + *Lb. helveticus* co-culture, AA was observed after 14 days of culture at a concentration of 0.02 g·100 mL^−1^. The production of AA in this case can be linked to citrate metabolism carried out by LAB. After 14 days of culture, the CA content decreased from 0.06 g·100 mL^−1^ to 0.00 g·100 mL^−1^ and 0.03 g·100 mL^−1^ for the *K. xylinus* + *Lb. delbrueckii* and *K. xylinus* + *Lb. helveticus* co-cultures, respectively (Table 2). CA naturally occurs in AW; therefore, in our study, its content did not change significantly in all monocultures and the *K. xylinus* + *Lb. acidophilus* co-culture, remaining at approximately 0.05–0.07 g·100 mL^−1^.

The content of GA in all mono- and co-cultures at the start of culture ranged from 0.05 to 0.08 g·100 mL^−1^. The presence of GA in AW was likely due to the addition of calcium gluconate to milk during the production of acidic cheeses, with its residues entering the AW (Pawlos et al. 2023). The initial LA content in all cultures ranged from 0.45 g·100 mL^−1^ to 0.53 g·100 mL^−1^. After 14 days, the lowest LA content was observed in the *K. xylinus* + *Lb. delbrueckii* co-culture (0.28 g·100 mL^−1^), and the highest was in the *K. xylinus* + *Lb. helveticus* co-culture (1.47 g·100 mL^−1^)*.* In the *K. xylinus* monoculture, after 3 days of cultivation, the LA content in the medium decreased to 0.44 g·100 mL^−1^, which could be the result of LA uptake by bacteria in the early growth phase, as reported previously by Matsuoka et al. (1996). It is also worth noting that a 3-day delay in BC biosynthesis (compared to co-cultures) was observed in the *K. xylinus* monoculture, which may indicate a significant link between LA uptake, energy production, and BC biosynthesis. However, the LA content increased over time, and after 14 days, it amounted to 1.04 g·100 mL^−1^. An increase in LA content during BC biosynthesis in the *K. xylinus* monoculture was also observed in the study by Jiang et al. (2023), where 2.41 g·100 mL^−1^ was obtained after 5 days of culture. This result was considered positive by the authors because the appropriate LA content may promote BC synthesis by accelerating the TCA cycle and gluconeogenesis, as well as directly affect the activity of enzymes involved in BC biosynthesis (Jiang et al. 2023).

#### Assessment of exopolysaccharides production capacity by LAB

**Fig. 3:**
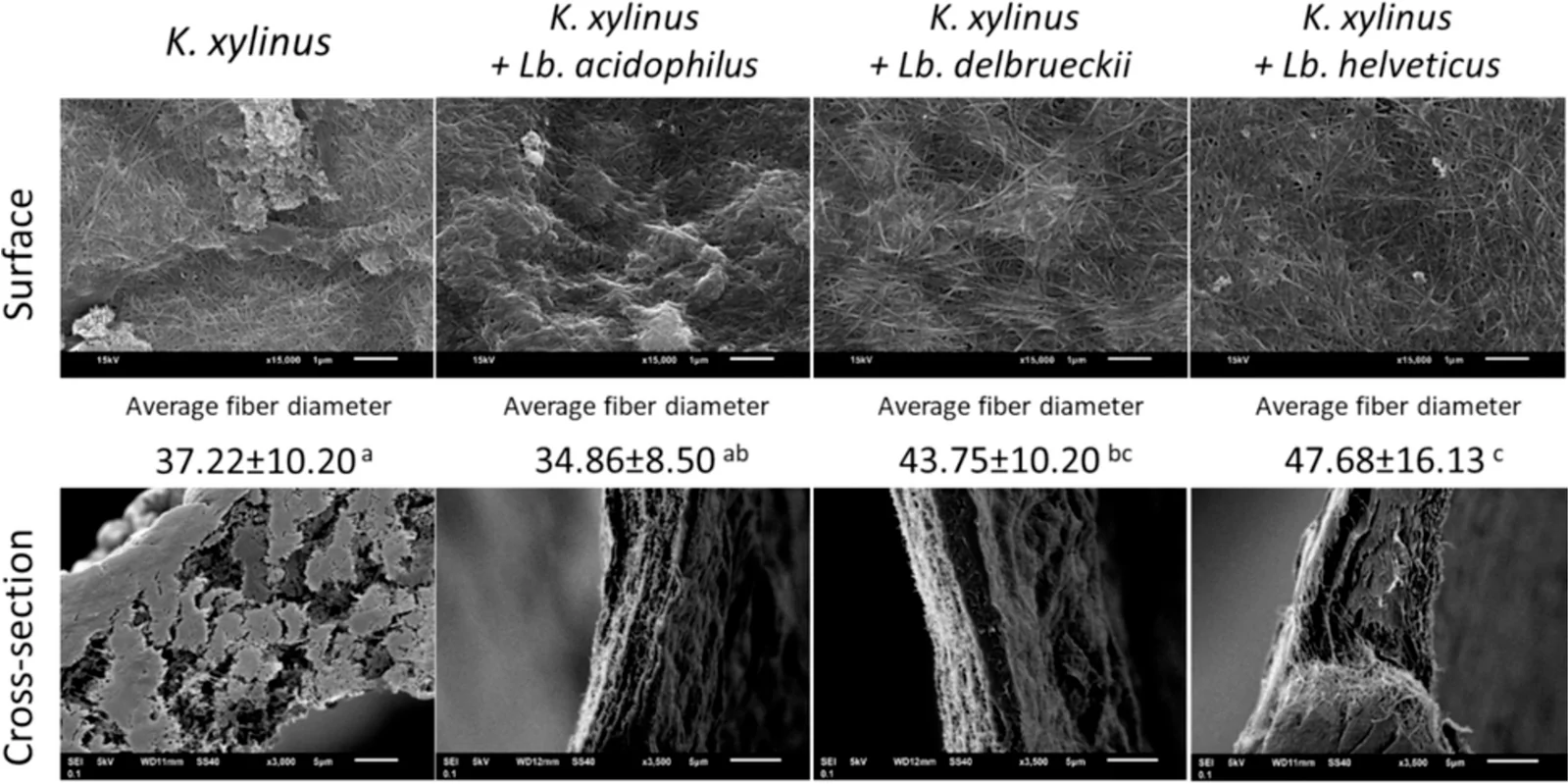
SEM images of surface and cross section of BC samples from chosen mono- and co-cultures. ^a–c^Means marked with the same superscript letters are not statistically significantly different at *α* = 0.05. High-resolution SEM images have been included in the supplementary materials

The EPS production capacity of LAB was assessed in two media: MRS medium dedicated to the growth of lactobacilli and in AW. In both media, it was noted that the *Lb. delbrueckii* strain produced the most EPS, while the *Lb. acidophilus* and *Lb. helveticus* strains were characterized by similar EPS production. In the MRS medium, the *Lb. delbrueckii* strain produced five times more EPS than in AW (1.04 g·L^−1^ and 0.24 g·L^−1^, respectively), while *Lb. acidophilus* and *Lb. helveticus* produced two times more EPS in the MRS medium (0.4 g·L^−1^ and 0.48 g·L^−1^, respectively) than in AW (0.20 g·L^−1^ and 0.21 g·L^−1^, respectively) (Table 3). In co-cultures, the level of EPS produced by LAB was comparable to that from LAB monocultures (except for the co-culture with *Lb. acidophilus*). This may suggest that co-culture with *K. xylinus* had no effect on the EPS production capacity of LAB (except *Lb. acidophilus*). Statistically significantly (*p* < 0.05), the highest yield of EPS was observed in the co-cultures of *K. xylinus* + *Lb. delbrueckii* and *K. xylinus* + *Lb. helveticus*. These differences in EPS content in the medium correspond to the average fiber diameter (Fig. 3) and the stress at break (Fig. 6) of BC obtained in the corresponding co-cultures. However, it should be noted that all LAB strains have been proven to produce EPS, which could affect the biosynthesis of BC, its extracellular release, and the formation of 1,4-β-glucan chains (Liu and Catchmark 2018).

**Table 3:** Exopolysaccharides produced by LAB in monocultures and co-cultures (g·L^−1^)

Strain	MRS medium (g·L^−1^)	AW medium (g·L^−1^)
*Lb. acidophillus* ATCC 4356	0.40 ± 0.11^a^	0.20 ± 0.01^a^
*Lb. delbrueckii* subs. *lactis* ATCC 4797	1.04 ± 0.20^b^	0.24 ± 0.01^a^
*Lb. helveticus* LhB01	0.48 ± 0.18^a^	0.21 ± 0.08^a^
*K. xylinus* + *Lb. acidophilus*	-	0.09 ± 0.02^a^
*K. xylinus* + *Lb. delbrueckii*	-	0.21 ± 0.02^b^
*K. xylinus* + *Lb. helveticus*	-	0.17 ± 0.03^b^

^a–b^Means marked with the same superscript letters in the same column are not statistically significantly different at *α* = 0.05

#### Surface characteristics and fibre arrangement in BC

The surface morphology, average fiber diameters**,** and fiber structure of cellulose obtained in *K. xylinus* monoculture and co-cultures are shown in Fig. 3.

Based on the SEM images, it was observed that the BC surface from all culture variants showed a reticular structure consisting of densely spaced, ultra-thin cellulose fibrils, forming a three-dimensional web. BC from the *K. xylinus* monoculture and from the *K. xylinus* + *Lb. acidophilus* co-culture were characterized by similar surface morphology with a small number of compact spaces between the fibers. Numerous spaces were observed on the surface of BC from the *K. xylinus* + *Lb. helveticus* co-culture, which were a result of loosely arranged BC fibers. In other studies, it was proven that a higher concentration of ascorbic acid in the medium resulted in lower content and greater porosity of BC (Raiszadeh-Jahromi et al. 2020). Raiszadeh-Jahromi et al. (2020) explained that organic acids can disturb the BC matrix, causing cracks and destructions in the homogeneity of its structure. In our study, the presence of LA and GA likely influenced the loosening of the BC fibre structure obtained in the *K. xylinus* + *Lb. helveticus* co-culture, as the medium from this co-culture was characterized by the highest content of these acids compared to other cultures (Table 2). In the SEM images of the cross section of cellulose (Fig. 3), it can be seen that the structure of BC from the *K. xylinus* monoculture was more compact, with an irregular arrangement of fibers and the space between them. In contrast, a layered arrangement of fibers was visible in the structure BC from co-cultures.

The average diameters of the cellulose fibers obtained in these studies were similar to the diameter of BC fibers (from 35 to 55 nm) characterized by other authors (Wang et al. 2018; Raiszadeh-Jahromi et al. 2020; Brugnoli et al. 2023a). The BC fibers from the *K. xylinus* monoculture (37.22 nm) were statistically significantly (*p* < 0.05) thinner than BC fibers from *K. xylinus* + *Lb. delbrueckii* co-culture (43.75 nm) and *K. xylinus* + *Lb. helveticus* co-culture (47.68 nm), which were connected and branched. No differences were observed in the diameter of BC fibers between BCs from *K. xylinus* monoculture and *K. xylinus* + *Lb. acidophilus* co-culture (34.86 nm), which were the thinnest. Statistically significantly, the thickest fiber diameter was characterized by BC from the *K. xylinus* + *Lb. helveticus* co-culture. Statistically significant differences in fiber diameter coincide with differences in stress at break (Fig. 6B). A similar result was obtained in the study by Liu and Catchmark (2019), in which the BC fibers obtained in the *Ga. hansenii* co-culture from *E. coli* were thicker than those from the *Ga. hansenii* monoculture and were locally connected into larger ribbons. It was hypothesized that EPS produced by *E. coli* could coat the surface of the co-crystallized BC microfibrils, which promoted their merging.

#### Chemical characterisation of bacterial cellulose

**Fig. 4:**
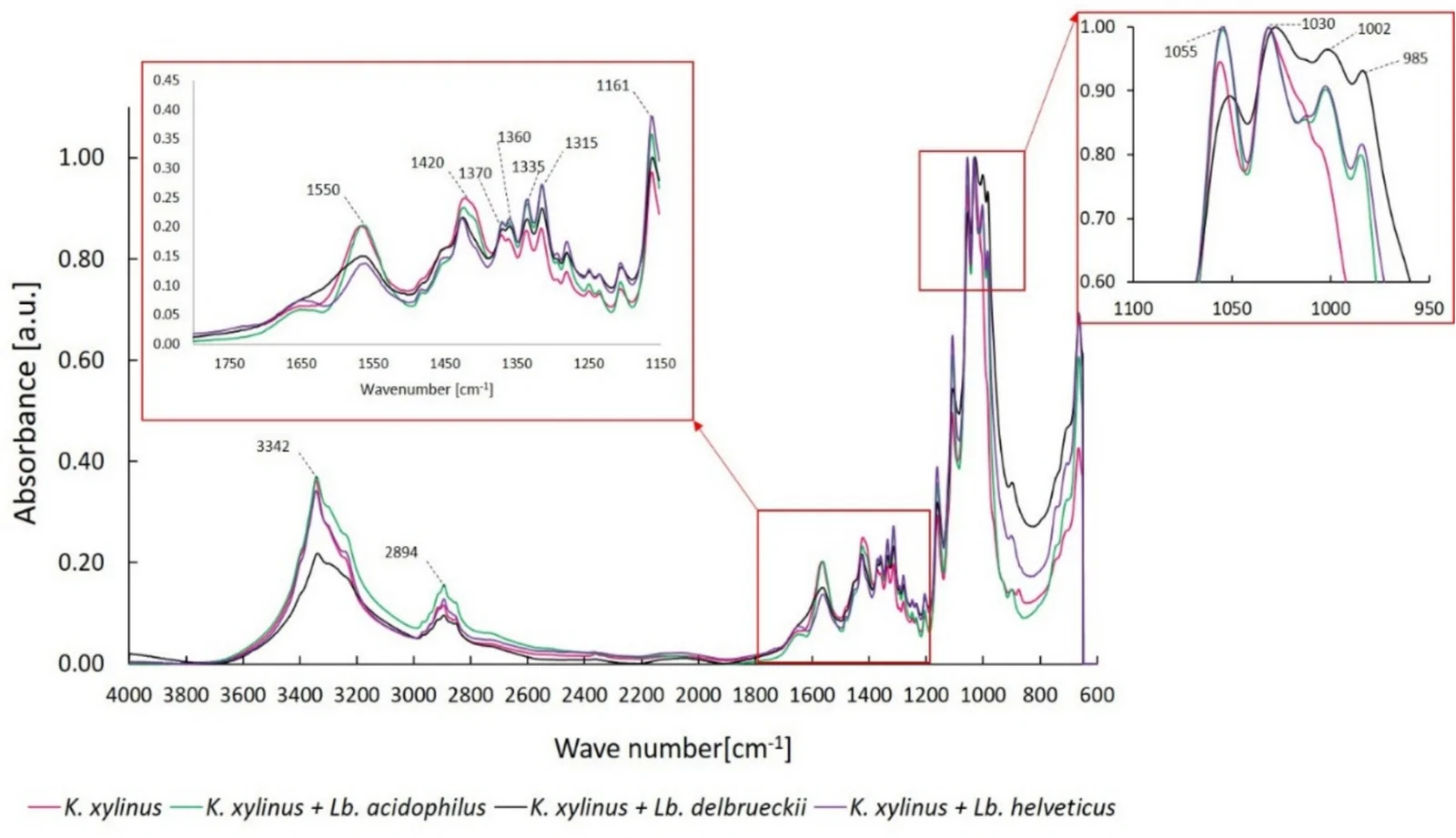
FTIR spectra of BC obtained in mono- and co-cultures

FTIR spectra of BC obtained in *K. xylinus* monoculture and co-cultures are shown in Fig. 4.

Characteristic peaks corresponding to the –OH stretching vibrations and H–O–H bending vibrations of absorbed water were observed around wavenumbers 3340 cm^−1^ and 1650 cm^−1^, respectively (Fig. 4). The peaks around 2890 cm^−1^ were related to C–H stretching vibrations in the CH_2_ and CH_3_ groups (Jiang et al. 2023). The signals at 1425 cm^−1^, 1160 cm^−1^, 1108 cm^−1^, 1055 cm^−1^, and 1030 cm^−1^ were assigned to the CH_2_ symmetric bending, C–O or C–C stretching vibrations, and C–O–C pyranose ring skeletal vibrations, respectively. Differences were observed in the intensities of some peaks, e.g., around wavenumber 1550 cm^−1^. A peak at ~ 1550 cm^−1^ likely corresponded to carboxylate ions. In the spectra of BC from the *K. xylinus* monoculture and the *K. xylinus* + *Lb. acidophilus* co-culture, this peak was significantly higher than in the spectra of BC from the *K. xylinus* + *Lb. delbrueckii* and *K. xylinus* + *Lb. helveticus* co-cultures.

### Thermal properties of BC

**Fig. 5:**
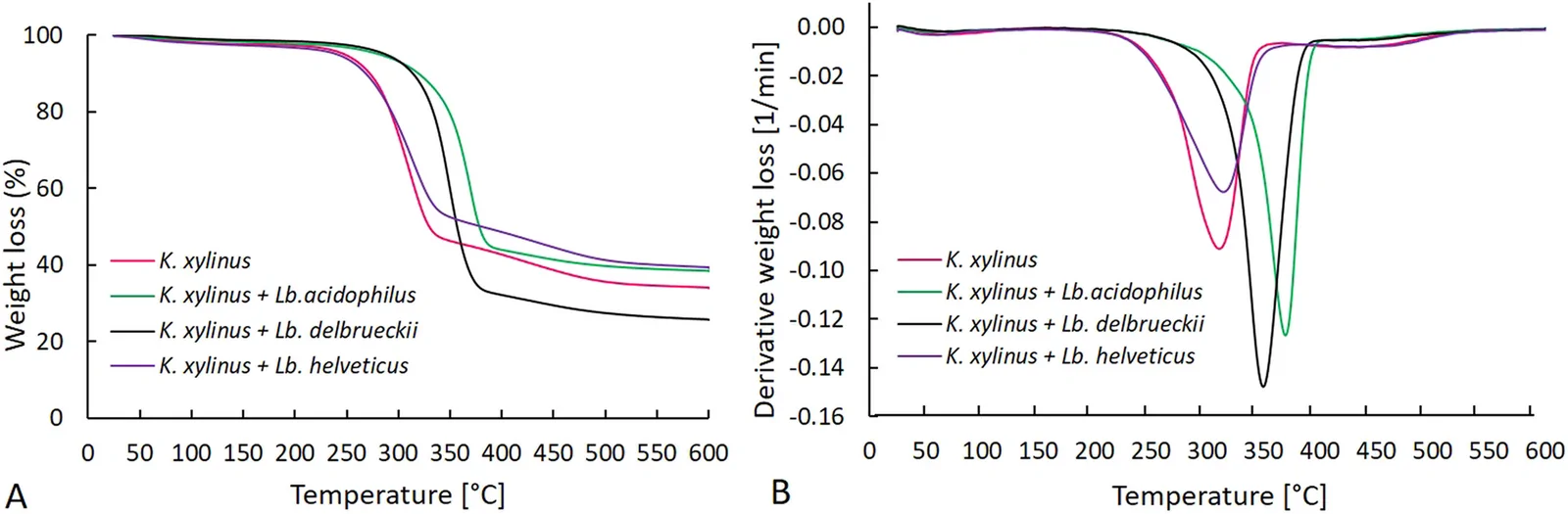
TGA (**A**) and dTG (**B**) curves of BC obtained in mono- and co-cultures

The TGA and DTG curves (Fig. 5) showed differences in the thermal properties of BC produced in the *K. xylinus* monoculture and co-cultures. Three steps of BC degradation under the influence of temperature were identified. In the first phase (25–120 °C), there was a mass loss due to water evaporation. Up to 250 °C, the samples were thermally stable. The greatest degradation of BC was observed in the second stage (temp. 120 to 400 °C). Specifically, 55% of BC from the *K. xylinus* monoculture, 55% of BC from the *K. xylinus* + *Lb. acidophilus* co-culture, 67% of BC from the *K. xylinus* + *Lb. delbrueckii* co-culture, and 49% of BC from the *K. xylinus* + *Lb. helveticus* co-culture were degraded at this stage. The decrease in the BC mass in this phase was caused by depolymerization and pyrolytic decomposition of glucose (Bagewadi et al. 2023). Kumar et al. (2021) found that 43% of BC from AW degraded at 250–415 °C, which is similar to our results. After the third stage of degradation (temp. 600 °C), 66% of BC from the *K. xylinus* monoculture degraded. BC from the *K. xylinus* + *Lb. helveticus* and *K. xylinus* + *Lb. acidophilus* co-cultures degraded to a similar degree, with 61% and 62% of the compound broken down, respectively. In the case of the *K. xylinus* + *Lb. delbrueckii* co-culture, 74% of BC degraded.

The maximum degradation temperature determining the maximum rate of degradation temperature (MRDT) of BC from the *K. xylinus* monoculture was about 303 °C, from the *K. xylinus* + *Lb. helveticus* co-culture about 307 °C, from the *K. xylinus* + *Lb. delbrueckii* co-culture about 342 °C, and from the *K. xylinus* + *Lb. acidophilus* co-culture about 361 °C (Fig. 5B).

### Mechanical properties

**Fig. 6:**
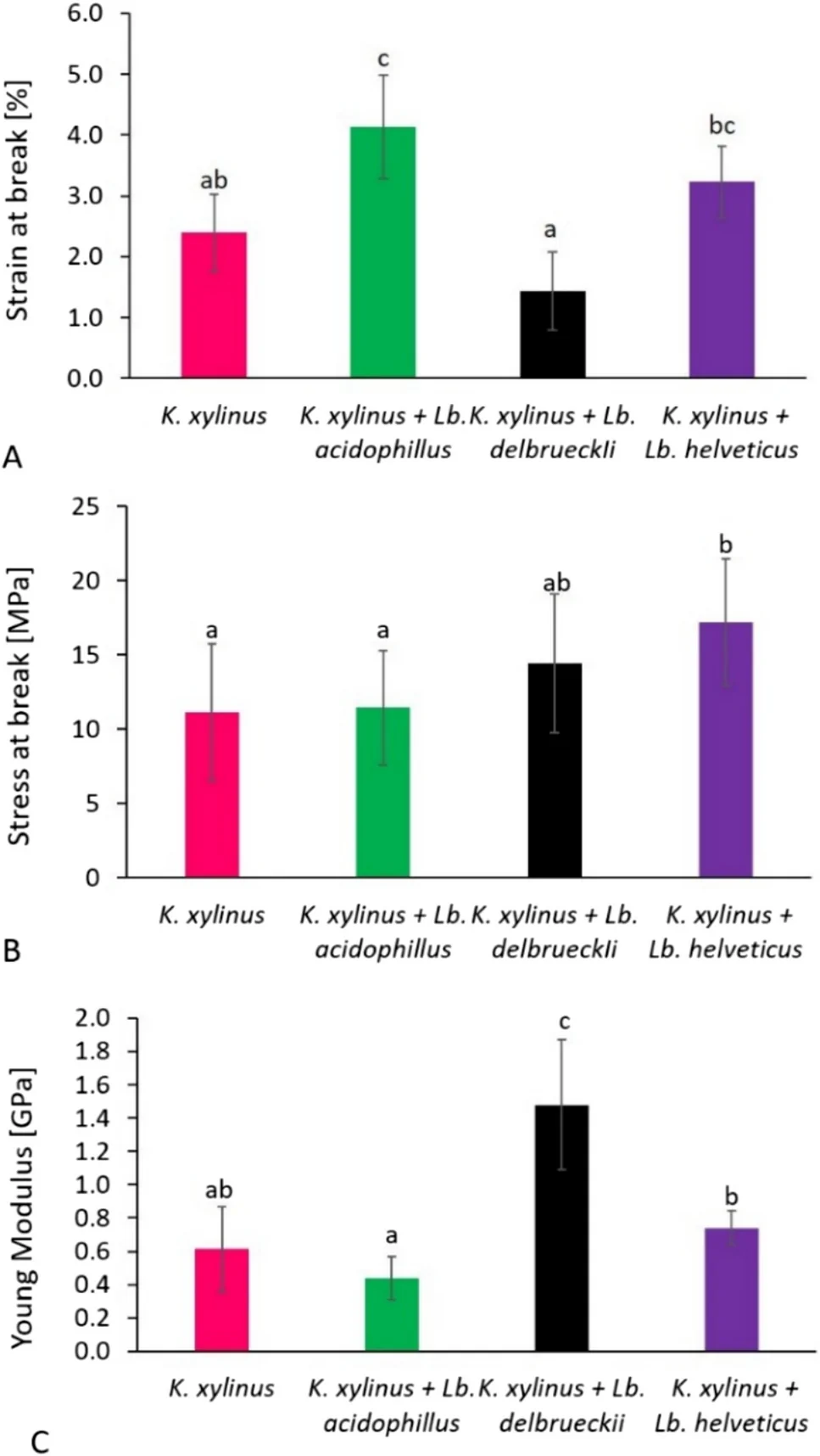
Mechanical properties of BC obtained in mono- and co-cultures, strain at break (**A**), stress at break (**B**), Young’s modulus (**C**); ^﻿a–c^Means marked with the same superscript letters are not statistically significantly different at α = 0.05﻿

In this study, the mechanical properties of BC obtained in monoculture and co-culture including strain at break, stress at break, and Young’s modulus were characterized (Fig. 6). To validate the obtained results and minimize their randomness, the mechanical properties of BC were tested in ten repetitions. Although the standard deviations for the averages obtained are high, they still remain typical for the analysis of BC mechanical properties (Liu and Catchmark 2019; Li et al. 2020).

This study showed that cellulose obtained from *K. xylinus* + *Lb. acidophilus* co-culture had the highest strain at break (Fig. 6A), which was statistically significantly different (*p* < 0.05) from BC obtained from *K. xylinus* monoculture and BC obtained from *K. xylinus* + *Lb. delbrueckii* co-culture. Stress at break BC obtained from *K. xylinus* + *Lb. acidophilus* and *K. xylinus* + *Lb. delbrueckii* co-cultures was similar to stress at break BC from *K. xylinus* monoculture. The highest value of this parameter was achieved by BC produced in the co-culture of *K. xylinus* + *Lb. helveticus*. BC from the *K. xylinus* + *Lb. delbrueckii* co-culture had the highest Young’s modulus, which was 1.48 GPa and was statistically significantly higher than the other BC samples.

## Discussion

In this study, the most BC was produced in *K. xylinus* + *Lb. acidophilus* and *K. xylinus* + *Lb. helveticus* co-cultures. The sudden increase in BC weight between days 12 and 14 in the *K. xylinus* + *Lb. acidophilus* and *K. xylinus* + *Lb. helveticus* co-cultures may be attributed to increased lactose consumption at this time (Fig. 2A and E). The stabilization of lactose levels in *Lb. acidophilus* and *Lb. helveticus* monocultures after 12 days of culture (Fig. 2A and E) suggests that these bacteria no longer consume lactose from the medium and convert it to LA after this time. This is confirmed by the constant pH and LA content in these monocultures after 12 days (Tables 1 and 2). In contrast, in the *K. xylinus* monoculture, lactose is still consumed between days 12 and 14 (Fig. 2A, C, E). Thus, it can be assumed that *K. xylinus* is responsible for lactose consumption after 12 days in co-cultures of *K. xylinus* + L*b. acidophilus* and *K. xylinus* + *Lb. helveticus*. Based on this, it can be hypothesized that *Lb. acidophilus* and *Lb. helveticus* in co-cultures consume lactose until day 12, after which, due to the disappearance of competition for the carbon source, *K. xylinus* becomes more active and utilizes it for increased BC production.

The positive effect of co-culture on BC biosynthesis was noted in studies by Seto et al. (2006), in which *Gluconacetobacter xylinus* was cultivated with *Lactobacilus mali*. Specifically, 4.2 g·L^−1^ of BC was obtained compared to 1.4 g·L^−1^ of BC produced in a *Ga. xylinus* monoculture. As a possible explanation for this phenomenon, the authors suggested an increased activity of enzymes responsible for sucrose metabolism and EPS production by LAB, though this was not proved experimentally. Additionally, attention was drawn to the fact that it is not just EPS, but EPS-producing LAB that promote cellulose production, facilitating physical interaction between cells. Cell coaggregation may support the assembly of BC fibers into matrices and facilitate interactions between cells at the quorum sensing (QS) level (Seto et al. 2006). QS is a phenomenon of chemical communication between bacterial cells, consisting of the synthesis and secretion of signaling molecules into the environment, which participate in regulating various physiological processes (Liu et al. 2018). The effect of QS on increased BC biosynthesis may be associated with the transcriptional regulatory protein LuxR, the increased expression of which has been noted in the co-culture of *K. nataicola* Q2 and *Lb. fermentum* SR. QS can also regulate the expression of *bcs* genes (encoding cellulose synthase) by regulating the level of cyclic diguanylate, which affects the formation of β−1,4glucan chains and the crystallization of microfibrils (Liu et al. 2018; Jiang et al. 2023). The *K. xylinus* UMCC 2756 strain contains the *luxR* gene; however, further research is required to confirm this mechanism (Gullo et al. 2019).

Increased BC biosynthesis was also observed in co-cultures of AAB with other microorganisms, e.g., with *Lactobacilus fermentum*, *Bacilus cereus*, and *Escherichia coli* (Liu and Catchmark 2019; Jiang et al. 2023; Li et al. 2023). The mechanisms promoting BC production in different co-cultures may vary. In one scheme, metabolites of one strain increase the activity of enzymes involved in ATP synthesis in AAB, thereby increasing energy levels and promoting cell growth (Li et al. 2023). The second mechanism presumably involves one strain assimilating a compound or metabolite that inhibits a certain metabolic pathway in the second strain. Reducing the content of the inhibitor causes this pathway to be activated. For example, in an *E. coli* co-culture, it assimilates GA, preventing GA from inhibiting the pathway responsible for BC biosynthesis in acetic bacteria (Liu and Catchmark 2019).

Compared to the monoculture, the consumption of lactose in *K. xylinus* + *Lb. acidophilus* and *K. xylinus* + *Lb. helveticus* co-cultures was almost twice as high. Nevertheless, when analyzing these two co-cultures, a certain correlation can be observed. In both the *K. xylinus* + *Lb. acidophilus* and *K. xylinus* + *Lb. helveticus* co-cultures, there was a significant (mild in the case of the co-culture with *Lb. helveticus*) decrease in the lactose content of the medium after 6–9 days of culture, but this did not reflect a significant increase in BC mass (Fig. 1) during this time. A potential explanation for this phenomenon is that lactose consumption could be related to the dominance of *Lb. acidophilus* and *Lb. helveticus*, which assimilated lactose and used it for their own metabolism, e.g., LA or EPS production. This may be confirmed by the observation that in the *K. xylinus* monoculture, the lactose consumption on days 6–9 is lower than the lactose consumption in the *Lb. acidophilus* and *Lb. helveticus* monocultures at this time. Moreover, lactose consumption by *Lb. acidophilus* and *Lb. helveticus* was observed until the 12th day of culture, after which time the lactose content of the medium with *Lb. acidophilus* and *Lb. helveticus* monocultures did not decrease (Fig. 2A). Assuming that the metabolism of lactose by *Lb. acidophilus* and *Lb. helveticus* proceeds similarly in co-cultures with *K. xylinus*, it can be assumed that after 12 days of culture the remaining lactose in the medium becomes easily available to *K. xylinus*, since they no longer have to compete with LAB for this sugar. Therefore, *K. xylinus* may have increased BC production only after 12 days of culture. Low lactose consumption (0.3 g·100 mL^−1^) in the *K. xylinus* + *Lb. delbrueckii* co-culture may suggest that allelopathic interactions occurred between *K. xylinus* and *Lb. delbrueckii*, meaning the growth and activity of one strain may have been limited by metabolites produced by the other strain. One possible explanation for this effect is that LAB produce compounds with high antioxidant activity (Xing et al. 2015; Zhang et al. 2017; Antolak et al. 2021). Antioxidants could negatively affect aerobic *K. xylinus* bacteria, in which the oxidative phosphorylation of sugars is one of the primary mechanisms for obtaining energy (He et al. 2022). Thus, *K. xylinus*, stimulated to obtain energy through alternative pathways, activated the substrate phosphorylation from non-sugar carbon sources by TCA (Zhong et al. 2013), which would explain the low consumption of lactose, the decreasing content of LA and CA, and the production of AA (Table 2). Another explanation suggests the inhibitory effect of metabolites (aldehydes, ketones, organic acids) of *K. xylinus* on the growth of *Lb. delbrueckii*, as evidenced by the lower content of LA, the main metabolite of LAB, compared to the *Lb. delbrueckii* monoculture. The impact of mutual interactions in the AAB co-culture with LAB was also noted by Xia et al. (2022). In the co-culture of *Acetobacter pasteurianus* with *Lb. helveticus*, inhibited growth of *Lb. helveticus* was observed compared to the monoculture, while the growth of *A. pasteurianus* remained unchanged. The authors argued that this was related to a higher content of AA in the medium, which could inhibit the growth of *Lb. helveticus*.

One of our goals in this study was to check the possibility of producing BC in AW where the main carbon source is lactose. According to literature data, one of the challenges associated with obtaining BC from AW is the low ability of AAB to assimilate whey proteins and uptake lactose. This is due to the absence of the *lacZ* gene in the genomes of most AAB, the expression of which determines the synthesis of β-galactosidase, the enzyme responsible for breaking down lactose into glucose and galactose (Kolesovs and Semjonovs 2020). Brugnoli et al. (2023a) used cheese whey as a medium for BC biosynthesis using, among others, *K. xylinus* UMCC 2756 and obtained more BC than in Hestrin-Schramm medium. However, before culturing, the whey was treated with β-galactosidase from *Aspergillus oryzae*, which confirms that *K. xylinus* UMCC 2756 is not able to degrade lactose into glucose and galactose (Brugnoli et al. 2023a). However, our research has shown that in the *K. xylinus* UMCC 2756 monoculture, the lactose content decreased, suggesting that probably it is included in some metabolic pathway of *K. xylinus* UMCC 2756, which enables BC production in AW. It is worth noting that the use of lactose and BC biosynthesis increased in the presence of LA in the medium and at pH 3.5–3.8. This observation is consistent with reports by Li et al. (2023), which showed that glucose consumption increased after medium supplementation with acetoin. Therefore, to increase BC production in pure AW, it is necessary to use other stimulants like LA, as its biosynthesis in AW is not very efficient. Another hypothetical explanation for the lactose consumption by *K. xylinus* is its oxidation to lactobionic acid (4-*O*-β-galactopyranosyl-D-gluconic acid, LBA) (Kiryu et al. 2019). LBA is formed as a result of enzymatic oxidation of lactose by the periplasmic membrane–bound quinoprotein glucose dehydrogenase (m-GDH). The presence of m-GDH and its oxidative activity has been proven in 48 AAB strains, with the highest activity found in *Komagataeibacte*r (formerly *Gluconacetobacter*) *medellinensis* NBRC3288 (Kiryu et al. 2015). Interestingly, the presence of the *gcd* gene was found in the genome of *K. xylinus* UMCC 2756 (GeneBank no. QQBI00000000) (Gullo et al. 2019), which encodes the production of m-GDH. However, these studies did not examine the content of LBA, so further research is needed to confirm the ability of *K. xylinus* UMCC 2756 to oxidize lactose.

In recent years, the possibility of obtaining BC in media with lactose as the primary carbon source has been studied, but the efficiency was unsatisfactory (Cielecka et al. 2021b). In studies on the cultivation of the *Komagataeibacter* sp. W1 strain by Wang et al. (2018), only 0.04 g·L^−1^ of BC was obtained, and during the cultivation of the *G. hansenii* GH1/2008 strain by Bolgova et al. (2023), only 0.1 g·L^−1^ was produced. In contrast, Singhsa et al. (2018) examined five strains of *K. xylinus* and found that the amount of BC produced from lactose may differ not only due to the strain used but also due to the cultivation method (stationary or mixed). Under stationary conditions, the highest BC production (approx. 1.4 g·L^−1^) from lactose was achieved by the *K. xylinus* strain, whose efficiency in shaken cultures did not change. In the stationary cultivation of the *K. xylinus* K1011 and *K. xylinus* K975 strains, 0.9 g·L^−1^ and 1.3 g·L^−1^ of BC was obtained, respectively, while in the mixed cultivation, the efficiency increased to approximately 4.7 g·L^−1^ and 2.4 g·L^−1^, respectively (Singhsa et al. 2018). Another example of one of the few studies showing the natural ability of AAB to assimilate lactose (and thus increase BC biosynthesis) is the research by Revin et al. (2018) which demonstrated that the *Gluconobacter sucrofermentans* B-11267 strain could assimilate lactose from whey and produced 5.45 g·L^−1^ of BC. A summary of research showing the possibilities of producing BC from AW as a standalone medium or an additive to other media is described in our previous publication (Płoska et al. 2023b).

**Fig. 7:**
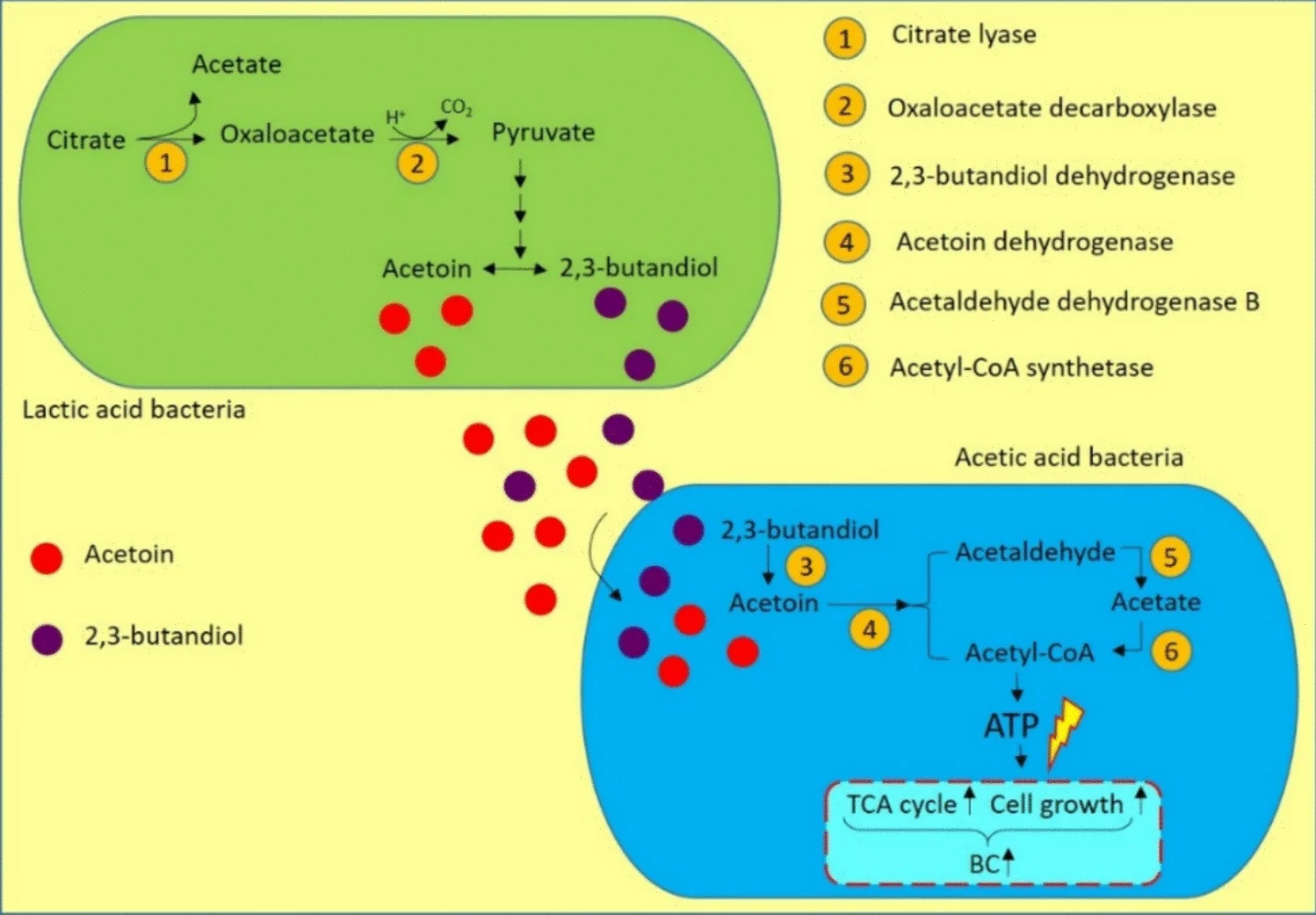
Hypothetical scheme of metabolite flow in acetic-lactic co-culture using citrate and acetoin metabolism as an example

A characteristic feature of microbial co-cultures is that the metabolites of one microorganism can be substrates entering the metabolic pathways of another one. Metabolism of citrate to acetate is typical of LAB (Oliveira et al. 2012). In homofermentative LAB, the production of acetate and oxaloacetate from citrate is catalyzed by citrate lyase (Fig. 7). The acetate is excreted outside the cell, and the oxaloacetate is decarboxylated to pyruvate by oxaloacetate decarboxylase. In subsequent steps, pyruvate may be further converted to lactate, acetate, formate, ethanol, or four-carbon aromatic compounds such as acetoin and 2,3-butanediol (Quintans, et al. 2008; Pudlik and Lolkema 2011; Bintsis 2018). The latter two metabolites may be relevant in the context of the AAB co-culture. According to Li et al. (2023), acetoin and 2,3-butanediol can positively affect the growth and activity of AAB by increasing the activity of acetoin dehydrogenase, which catalyzes the conversion of acetoin to acetyl-coenzyme A (CoA) and acetaldehyde. Additionally, acetaldehyde may be converted to acetate and then acetyl-CoA by acetaldehyde dehydrogenase and acetyl-CoA synthetase, respectively. An increase in acetyl-CoA concentration leads to the generation of more ATP molecules, which in turn provides the energy necessary for cell growth and BC biosynthesis (Fig. 7). This mechanism was confirmed in the co-culture of *K. xylinus* with *Bacillus cereus*, where acetoin and 2,3-butanediol were metabolites of *B. cereus*. Notably, 4.4 g·L^−1^ of BC was obtained from the co-culture, compared to 1.2 g·L^−1^ from the *K. xylinus* monoculture (Li et al. 2023). A hypothetical scheme illustrating the effect of LAB metabolites on AAB metabolism using citrate and acetoin metabolism as example is shown in Fig. 7.

In AAB, gluconic acid and its derivatives are formed as a result of aerobic glucose metabolism, where they participate in one of two pathways for energy production in the cell. When glucose is the carbon source in the medium, it is phosphorylated by glucokinase to glucose-6-phosphate, which is then converted to 6-phosphogluconolactone. This process is associated with the reduction of nicotinamide adenine dinucleotide phosphate (NADP^+^) to NADPH, generating energy and transferring electrons to electron acceptors (Liu et al. 2018). In contrast, when glucose is absent from the medium and another carbon source, such as mannitol, lactose, or fructose, is present, GA is produced in very low concentrations or not produced at all (Anguluri et al. 2022). In our study, the primary carbon source in AW was lactose, which justifies the low content of GA. In almost all cultures, the GA content decreased over time, with the exception of the *K. xylinus* + *Lb. helveticus* co-culture, where the GA content increased. The decrease in GA content in the medium can be explained by the hypothesis put forward by Liu et al. (2018), who argued that in the absence of glucose in the medium, GA can be converted to glucose-6-phosphate and further used as a carbon source for cell growth and BC production via the pentose phosphate pathway. Interestingly, the increase in GA content in the *K. xylinus* + *Lb. helveticus* co-culture may be correlated with the increase in galactose content. This supports the hypothesis about the oxidation of lactose to LBA by *K. xylinus*. It is possible that *Lb. helveticus* produces enzymes that enable the hydrolysis of LBA to galactose and GA, thus explaining the observed increase in their content in the medium (Fig. 2F).

The FTIR spectra of BC obtained in the *K. xylinus* monoculture and acetic-lactic co-cultures were similar (Fig. 4). In all samples, peaks corresponding to bonds and functional groups characteristic of BC were observed, and no new peaks were observed in spectra of BC from co-cultures. In studies by Liu and Catchmark (2019), the spectrum of BC from the *Ga. hansenii* and *E. coli* co-culture showed the presence of a peak corresponding to the C–N bonds in glucosamine, which was derived from EPS produced by *E. coli*. In our study, despite the proven ability of LAB to produce EPS (Table 3), no extra peaks indicating the occurrence of C–N bonds were observed. Nevertheless, from the wavenumber of 1400 to 600 cm^−1^ (Fig. 4), the peak intensity in the spectra of BC from all co-cultures was higher compared to that in the spectra of BC from the *K. xylinus* monoculture. Peak intensity is assumed to be directly proportional to the number (per unit volume) of functional groups and bonds occurring in the material (Farhadi and Sohbatzadeh 2023). Therefore, considering the similarity of functional groups and bonds in the chemical structure of BC and EPS, potential interactions between BC and EPS produced by LAB can be assumed. A significant difference in peak intensity in the FTIR spectra was observed in BC from the *K. xylinus* + *Lb. delbrueckii* co-culture. In this case, the intensity of the peak corresponding to the hydroxyl group (3336 cm^−1^) was lower than that seen in the spectra of other BC, which may indicate a lower number of hydroxyl groups and hydrogen bonds. According to Sharma et al. (2023), the intensity of the peak at wavenumber 3336 cm^−1^ indicates the strength of the intramolecular H3(O)H…O(5) bond, which can be increased in the presence of LA in the medium. This explains the lower intensity of this peak in the spectrum of BC from the *K. xylinus* + *Lb. delbrueckii* co-culture, where the lowest LA content was observed (Table 2) compared to BC from other co-cultures and monoculture. It can be assumed that minor variations in the FTIR spectra of BC may have been influenced by different combinations of strains and differences in metabolites in the co-culture system, as mentioned by other researchers (Jiang et al. 2023).

Based on the MRDT values, it was found that the co-cultures *K. xylinus* + *Lb. acidophilus* and *K. xylinus* + *Lb. helveticus* positively affected the thermal properties of BC, with the highest thermostability exhibited by BC obtained from the co-culture *K. xylinus* + *Lb. acidophilus*. The apparent shift in the maximum temperature of BC degradation from the *K. xylinus* monoculture and the *K. xylinus* + *Lb. helveticus* co-culture toward a lower temperature may be related to the low crystallinity of these BC variants and a higher share of the amorphous fraction (unpublished results) compared to BC from the other cultures. According to Cichosz and Masek (2020), among others, the degree of crystallinity and the ratio of crystalline to amorphous areas determine the thermal stability of cellulose. The more amorphous areas in the structure (lower crystallinity), the more easily it degrades. According to Lin et al. (2023), changes in BC crystallinity, and therefore thermostability, can be caused by various interactions in the culture medium during crystallization, such as increasing the viscosity of the medium. Additionally, based on the conducted research, we cannot definitively state that the co-culture product is pure BC. We can suspect that some part of the EPS produced by the co-cultured LAB may have built into the BC structure, but we do not have definite evidence for this. On the basis of our results (including unpublished ones) and based on literature data, we assume that the differences in, among others, the thermal stability of the polymer obtained are due to specific interactions between *K. xylinus* and the particular strain of LAB.

The mechanical properties of BC depend on many factors such as culture conditions, medium composition, fiber concentration, fiber arrangement, and bacterial strain used to obtain BC (Cielecka et al. 2021a; Brugnoli et al. 2025). Cellulose obtained from the *K. xylinus* + *Lb. acidophilus* and *K. xylinus* + *Lb. helveticus* co-ultures was characterized by the highest strain at break (Fig. 6A), which could be related to a higher LA content in the media and a lower pH in these culture variants. As already mentioned, the presence of LA in the medium may increase the strength of the intramolecular H3(O)H…O(5) bonds within the glucopyranose ring (Sharma et al. 2023). In turn, the created intra- and intermolecular bonds in cellulose affect not only the physical properties of the polymer, including solubility, hydroxyl reactivity, and crystallinity, but also play an important role in its mechanical properties (Fan et al. 2012). According to Sharma et al. (2023), the addition of LA to the medium significantly increased the strain at break of the produced BC compared to the BC obtained in the medium without LA. These results also confirm the reports by Cielecka et al. (2021b) where LA medium supplementation positively influenced the strain at break. Moreover, it was shown that the strain at break of BC obtained in media with glucose and at different pH varied significantly, which would indicate the influence of the pH of the medium on the mechanical properties of BC. In our research, this hypothesis can be confirmed by the difference between the high strain at break of BC from the *K. xylinus* + *Lb. acidophilus* and *K. xylinus* + *Lb. helveticus* co-cultures, where the final pH was 3.8 and 3.6, respectively (Table 1), and the low strain at break of BC obtained in the *K. xylinus* + *Lb. delbrueckii* co-culture, where the final pH was approx. 6.0. The effect of the pH of the medium can also be observed in the case of Young’s modulus (Fig. 6C); however, in this instance, a reverse trend was noted. The highest Young’s modulus was characterized by BC obtained in the *K. xylinus* + *Lb. delbrueckii* co-culture, and it was significantly (*p* < 0.05) higher than the values for other BC samples. Nevertheless, it is also worth noting that in the case of this co-culture, the high value of Young’s modulus could have been influenced by EPS produced by *Lb. delbrueckii* (Table 3). Liu and Catchmark (2018) conducted studies which demonstrated that the addition of EPS to the culture medium had a positive effect on the Young’s modulus of BC compared to BC obtained from the Hestrin-Schramm medium without EPS. Moreover, it was observed that depending on the concentration, the addition of EPS may have a different effect on the strain at break and the stress at break. In our study, the highest stress at break value (Fig. 6B) was observed for cellulose obtained in the *K. xylinus* + *Lb. helveticus* co-culture (17.18 MPa), and a slightly lower value was recorded for BC produced in the *K. xylinus* + *Lb. delbrueckii* co-culture (14.43 MPa)*.* These results can be linked to the arrangement and the largest diameter of BC fibers from these co-culture variants. It is possible that more force was required to tear apart thicker BC fibers, resulting in increased tensile stress (Cielecka et al. 2021a). Similar conclusions were formulated by Liu and Catchmark (2019) who, in research on the *Ga. hansenii* and *E. coli* co-culture, showed that the BC obtained from the co-culture was characterized by higher density and thicker fibers compared to BC from the *Ga. hansenii* monoculture, which affected its mechanical properties. On the other hand, Brugnoli et al. (2025) increased Young’s modulus and stress at break of the BC-hyaluronic acid composite obtained in co-culture of AAB with LAB, explained by high BC content and high crystallinity. Nevertheless, the authors noted the diverse effects of different strains on the mechanical properties of BC, which implies the necessity of proper selection of strains for co-culture.

## Conclusions

In this study, it was shown that AW, a side product of the dairy industry, can be a valuable and efficient substrate for the biotechnological production of BC both in AAB monoculture and in AAB-LAB co-culture. The efficiency of biosynthesis and its properties varied depending on the used variant of co-culture of *K. xylinus* and LAB. It was found that co-cultures of AAB with LAB exhibiting higher acidification capabilities, i.e., *Lb. acidophilus* and *Lb. helveticus*, promoted BC production more effectively than the co-culture with *Lb. delbrueckii* or the *K. xylinus* monoculture. This fact confirms the first part of our hypothesis, suggesting that in the AAB co-culture with LAB, BC biosynthesis in AW can be increased. The production of BC was influenced by the presence of LA in the medium, which enhanced the activity of enzymes with TCA, resulting in the production of more energy, which probably translated into the biosynthesis of BC. It can be assumed that in the *K. xylinus* + *Lb. delbrueckii* co-culture, negative allelopathy occurred, consisting in the growth and activity of one strain being limited by metabolites produced by the other strain. However, further research is necessary to learn the detailed mechanism of this relationship. It was observed that the *K. xylinus* UMCC 2756 strain used lactose derived from AW; however, the BC production took place only when LA was present in the medium and at a lower pH (3.5–3.8). This suggests that efficient BC biosynthesis from AW should be supported by additional LA supplementation.

The consumption of lactose from whey by *K. xylinus* UMCC 2756 despite the lack of the *lacZ* gene, encoding β-galactosidase, is a mechanism worth investigating at the molecular biology level. Here, it would be of particular interest to find and study a potential metabolic pathway in AABs in which lactose is incorporated, which would explain how AABs acquire a carbon source to build BC subunits. It can be assumed that lactose is oxidized to LBA. In this case, conducting molecular and bioinformatics analyses may be of particular interest because the *gcd* gene, which encodes m-GDH responsible for oxidizing lactose to LBA, was found in *K. xylinus*.

Our research confirmed the ability of LAB to produce EPS in AW. Notably, in AW, the EPS production was two and four times lower than in the MRS medium for *Lb. acidophilus*, *Lb. helveticus*, and *Lb. delbrueckii*, respectively. It was also demonstrated that LAB produce EPS in co-cultures, which could have influenced, among other factors, the properties of BC. By analyzing the FTIR spectra and basing on the similarity of functional groups and bonds in the chemical structures of BC and EPS, potential interactions between BC and EPS produced by LAB can be assumed. EPS presumably influenced the thickness and structure of BC fibers as well as their spatial arrangement. Increased thermostability was noted in BC obtained from the *K. xylinus* + *Lb. acidophilus* co-ulture compared to other BC samples. Cellulose obtained in co-cultures was characterized by better mechanical properties. The *K. xylinus* + *Lb. helveticus* co-culture positively influenced the stress at break of BC. BC from the *K. xylinus* + *Lb. delbrueckii* co-culture exhibited the highest Young’s modulus. In turn, BC obtained in the *K. xylinus* + *Lb. acidophilus* co-culture was characterized by the highest strain at break. This confirms the second part of our hypothesis that BC produced in the co-culture of AAB with LAB in AW shows improved chemical-hysical and mechanical properties.

Future study of the relationships discovered in this work may contribute to more effective biosynthesis of BC in AW by acetic-lactic co-cultures. It has been demonstrated that the use of microorganisms with different metabolic abilities in co-cultures may lead to the production of metabolites stimulating BC biosynthesis as well as increase the availability of nutrients present in the media. In turn, cultivating co-cultures in inexpensive by-product media, such as AW, can not only reduce the costs of producing BC but also decrease the negative impact of this by-product on the environment.

## Supplementary Information

Below is the link to the electronic supplementary material.

MOESM 1 (DOCX 10,789 KB)

## Data Availability

The datasets and materials from this study can be available upon request to interested researchers.
