# Supplementary material for: Study on obtaining bacterial cellulose by Komagataeibacter xylinus in co-culture with lactic acid bacteria in whey
Source: Appl Microbiol Biotechnol. 2025 Aug 21;109:191. doi: 10.1007/s00253-025-13582-3 (PMC12370864; doi:10.1007/s00253-025-13582-3)
Supplement: Supplementary file 1 [file 253_2025_13582_MOESM1_ESM.docx]

**SURFACE IMAGES**

*K. xylinus*

*K. xylinus + Lb. acidophilus*

*K. xylinus + Lb. delbrueckii*

*K. xylinus + Lb. helveticus*

**CROSS-SECTION IMAGES**

*K. xylinus*

*K. xylinus + Lb. acidophilus*

*K. xylinus + Lb. delbrueckii*

*K. xylinus + Lb. helveticus*
